# Hydrogen Sulfide Facilitates the Impaired Sensitivity of Carotid Sinus Baroreflex in Rats with Vascular Calcification

**DOI:** 10.3389/fphar.2017.00629

**Published:** 2017-09-12

**Authors:** Hui Li, Xu Teng, Rui Yang, Qi Guo, Hongmei Xue, Lin Xiao, Xiaocui Duan, Danyang Tian, Xiaohong Feng, Yuming Wu

**Affiliations:** ^1^Department of Physiology, Institute of Basic Medicine, Hebei Medical University Shijiazhuang, China; ^2^Hebei Key Lab of Laboratory Animal Science, Hebei Medical University Shijiazhuang, China; ^3^Key Laboratory of Vascular Medicine of Hebei Province Shijiazhuang, China; ^4^Hebei Collaborative Innovation Center for Cardio-Cerebrovascular Disease Shijiazhuang, China

**Keywords:** baroreflex, endoplasmic reticulum stress, hydrogen sulfide, perfusion of isolated carotid sinus, vascular calcification

## Abstract

Arterial baroreflex is a general mechanism maintaining cardiovascular homeostasis; its sensitivity is reduced in vascular calcification (VC). Hydrogen sulfide (H_2_S) treatment facilitates baroreflexive sensitivity in normal and hypertensive rats. Here, we aimed to detect the effect of H_2_S on baroreflexive sensitivity in rats with VC. The rat VC model was induced by vitamin D_3_ plus nicotine for 4 weeks. The sensitivity of baroreflex was detected by perfusing the isolated carotid sinus. VC was assessed by hematoxylin and eosin (H&E) staining, Ca^2+^ content and alkaline phosphatase (ALP) activity. Protein levels were detected by western blot analysis. Vitamin D_3_ plus nicotine induced structural disorder and elevated Ca^2+^ content in the aortic and carotid arterial wall and increased plasma ALP activity. In the calcified aorta and carotid artery, protein levels of contractile phenotype markers of vascular smooth muscle cells (VSMCs) were downregulated and that of osteoblast-like phenotype markers and endoplasmic reticulum stress (ERS) markers were upregulated. NaHS treatment ameliorated the histologic disorder and Ca^2+^ content in the calcified aorta and carotid artery, inhibited the elevated plasma ALP activity, and prevented the transformation of the VSMC phenotype and activation of ERS in rats with VC. Chronic NaHS treatment prevented the impairment of the baroreflex sensitivity and acute NaHS treatment dose-dependently improved the sensitivity in rats with VC. Our results suggested that H_2_S could directly facilitate the impairment of baroreflex in rats with VC and ameliorate VC, which might provide new target and strategy for regulation of the baroreflex and therapy of VC.

## Introduction

Ectopic calcification, calcium crystal deposition in soft tissues, is a universal vascular pathophenotype linked with aging, hypertension, atherosclerotic cardiovascular diseases, diabetes, and chronic kidney disease (Leopold, 2015). Vascular calcification (VC) forecasts impaired clinical results and crucial disadvantageous cardiovascular events, as demonstrated in a series of population-based investigations (Bild et al., 2005; Detrano et al., 2008; DeFilippis et al., 2011). In the last few decades, VC has been considered an active biological process similar to bone development that is highly regulated and preventable and can be reversed (Boström et al., 2011; Lanzer et al., 2014). Many endogenous active substances demonstrated to regulate VC via humoral regulation include hydrogen sulfide (H_2_S), adrenomedullin, intermedin, cortistatin, and insulin (Zhao and He, 1993; Cai et al., 2010a,b; Liu et al., 2010; Chang et al., 2013; Lanzer et al., 2014; Yang et al., 2016). However, the regulation effect of autonomic nerves is still largely unknown.

In the homeostatic mechanism arterial baroreflex, vascular, and cardiac function are modulated via detection of a change in tension in arterial vascular walls. The baroreflex arc consists basically of an afferent element, central neural element, and autonomic neuroeffector element. The baroreceptors are the major afferent elements of the reflex arc, which are sensors in the arterial walls mainly consisting of the aortic and carotid baroreceptor. The sensors detect the mechanical modification of arterial walls by afferent nervous terminals and trigger the excitation of these afferent nerves. The excitatory signal is then conveyed to the nucleus tractussolitary (NTS) in the dorsal medial of medulla oblongata. The NTS identifies the information from the baroreceptors, then produces the parasympathoexcitatory and sympathoinhibitory responses. Via the baroreflex, an increase in blood pressure causes reduced heart rate and stroke volume and enhanced vasodilatation, which restores blood pressure to normal levels (Benarroch, 2008). Baroreflex sensitivity is considered a comprehensive marker of the universal integrity of the autonomic nervous system (Robinson and Carr, 2002), which can be regulated by many endogenous active substances, such as angiotensin II and H_2_S (Zhang et al., 2015).

H_2_S is a colorless gas with a characteristic smell. Besides its well-known toxicity, H_2_S is now considered an endogenous gaseous signaling molecule in addition to nitric oxide and carbon monoxide and can be endogenously generated in mammalian tissues. Many studies have confirmed that H_2_S is a potential regulator of cardiovascular function and exerts a protective effect on the pathogenesis and progression of cardiovascular diseases, including hypertension, myocardial infarction, cardiac hypertrophy, and ischemic/reperfusion injury (Wang, 2012; Shen et al., 2015; Ueda et al., 2016). In addition, numerous studies have demonstrated that hydrogen sulfide is a vasodilator, that it decreases blood pressure acutely, that it acts as an anti-angiogenic and anti-inflammatory agent, and that cystathionine gamma lyase, a crucial endogenous enzyme contributed to production of H_2_S, knock-out mice are hypertensive (Yang et al., 2008; Yang and Wang, 2015).

Our previous investigations revealed that H_2_S facilitates baroreflexive sensitivity in normal rats by acting on the baroreceptor (Xiao et al., 2006; Guo et al., 2016) or the rostral ventrolateral medulla (Guo et al., 2011; Duan et al., 2015). Impaired baroreflexive sensitivity in rats with hypertension or diabetes can be rescued by H_2_S treatment (Gu et al., 2013; El-Sayed et al., 2016). However, the effect of H_2_S on baroreflex in rats with VC is unknown.

Reduced baroreflex sensitivity has been reported in patients with VC and chronic kidney disease (Chesterton et al., 2005), which may contribute to dysfunction of the cardiovascular system and increased mortality (McIntyre, 2007; Kaur et al., 2016). Therefore, rescued baroreflex sensitivity might ameliorate the cardiovascular dysfunction and mortality with VC. Considering the facilitating effect of H_2_S on baroreflex, H_2_S could rescue the impaired sensitivity of baroreflex in VC.

The VC in rats induced by vitamin D3 plus nicotine (VDN), firstly reported by Niederhoffer et al. (1997), is a representative model of medial calcification, which is the common vascular pathology in hypertension, diabetes, chronic renal failure, and aging. Vitamin D3 induced VC via stimulation of alkaline phosphatase and suppression of parathyroid hormone. The effect of nicotine was speculated to be related to release of catecholamine and artery constriction (Wallin et al., 2001). We presently used the VDN model and reperfusion of the isolated carotid sinus to investigate the effect of H_2_S on baroreflex sensitivity in VC.

## Materials and methods

### Animals and experimental procedure

Male Sprague–Dawley rats (180~200 g) were from the Animal Center, Hebei Medical University (Shijiazhuang, China) and were housed under standard conditions (room temperature 20 ± 8°C, humidity 60 ± 10%, lights from 6:00 to 18:00) with free access to standard rodent chow and water. All animal procedures complied with the Animal Management Rule of the Ministry of Health, People's Republic of China (document no. 55, 2001) and the National Institutes of Health guide for the care and use of Laboratory animals (NIH Publications No. 8023, revised 1978) and were approved by the Animal Care Committee of Hebei Medical University.

The chronic effect of H_2_S on baroreflex was investigated in rats randomly divided into 4 groups (*n* = 8 each) for treatment: control; sodium hydrosulfide (NaHS); calcification (Cal); and Cal + NaHS. VC in rats was induced by VDN in rats, and NaHS (56 μmol/kg, injected intraperitoneally daily for 4 weeks; Sigma, St. Louis, MO, USA) was an H_2_S donor. The groups without NaHS treatment were injected with the same dose of normal saline for 4 weeks. At the end of the 4 weeks, rats were prepared for perfusion of isolated carotid sinus, then blood, aortas and carotid arteries were collected for further detection.

The acute effect of H_2_S on baroreflex was investigated in rats with VC. After 4 weeks of feeding, rats with VC were prepared for perfusion of the isolated carotid sinus and randomly divided into 4 groups (*n* = 8 each) for treatment: Cal and Cal + NaHS (25; 50; and 100 μmol/L). Rats in the Cal group were perfused with Krebs-Henseleit solution, and rats in the last three groups were perfused with 25, 50, and 100 μmol/L NaHS for 1 h respectively.

### Preparation of VC model in rats

The VC model was induced by VDN as described (Niederhoffer et al., 1997) with modification. Male rats were given vitamin D_3_ (300,000 IU/kg, intramuscularly; Sigma) simultaneously with nicotine (25 mg/kg in 5 ml peanut oil, intragastrically; Sigma) at 8:00 on the first day. The nicotine administration was repeated at 20:00. On days 2 and 15, rats were re-treated with vitamin D3. Rats in the control group received an intramuscular injection of normal saline and 2 intragastrical administrations of peanut oil without nicotine (5 ml/kg).

### Perfusion of left isolated carotid sinus

The perfusion of isolated carotid sinus was as described (Wu et al., 2006; Zhou et al., 2012). Briefly, the carotid sinus areas were sufficiently revealed, and the superior laryngeal nerves, bilateral aortic nerves, right carotid sinus nerve, cervical sympathetic nerves and recurrent laryngeal nerves were all cut. The common, external and internal carotid arteries and smaller arteries branching from these vessels were ligated, carefully leaving the left carotid sinus nerve undisturbed. To exclude chemoreceptor activation, the occipital artery at its origin from the external carotid artery was ligated. One catheter inserted into the left carotid artery was an inlet tube, and another catheter inserted retrogradely into the external carotid artery was an outlet tube. Warm (37°C) oxygenated Krebs-Henseleit solution was used to perfuse the carotid sinus. The intrasinus pressure (ISP) was controlled by using a peristaltic pump. The ISP and blood pressure (BP) were simultaneously recorded on a polygraph (RM-6240; Chengdu Instrument Factory, Chengdu, China). Perfusion of the left carotid sinus with elevated ISP produced a functional curve of the ISP–BP relationship, and the functional baroreflex parameters threshold pressure (TP), saturation pressure (SP), equilibrium pressure (EP), peak slope (PS), reflex decrease of BP (RD), and operating range (OR) were calculated. TP was the ISP at which BP began to decrease in response to the increase of the ISP. SP was the ISP at which BP just showed no further reflex decreases with an increase in the ISP. OR was calculated as SP minus TP.

### Hematoxylin and eosin (H&E) staining

To detect morphologic changes with VC, the thoracic aorta and carotid artery were separated and embedded in 4% paraformaldehyde for H&E histopathological staining.

### Detection of calcium content and ALP activity

Calcium content was detected by using colorimetric kits (BioSino Biotechnology and Science, Beijing) by a reaction with o-cresolphtalein complexone, and plasma ALP activity was assessed by using an ALP colorimetric assay kit (BioSino Biotechnology and Science).

### Western blot analysis

The aorta and carotid artery were homogenized in lysis buffer (100 mg: 1 mL). Equal amounts of protein samples were loaded on 10% polyacrylamide gels, transferred to nitrocellulose membranes soaked in 5% non-fat dried milk for 1 h, then incubated serially with primary antibodies overnight at 4°C and with the secondary antibody for 1 h at room temprature. The reaction was visualized by enhanced chemiluminescence, and an autoradiograph was scanned. Protein concentrations were analyzed by using NIH ImageJ software and normalized to that of β-actin. All experiments were repeated at least three times.

### Statistical analysis

Statistical analysis involved use of GraphPad Prism v5.00 for Windows (GraphPad Software, San Diego, CA, USA). Two groups were compared by unpaired Student *t*-test and ≥3 groups by one-way ANOVA, then Newman–Keuls test. Data are expressed as mean ± SD. *p* < 0.05 was considered statistically significant.

## Results

### Chronic H_2_S treatment ameliorated VC in rats

**Figure 1:**
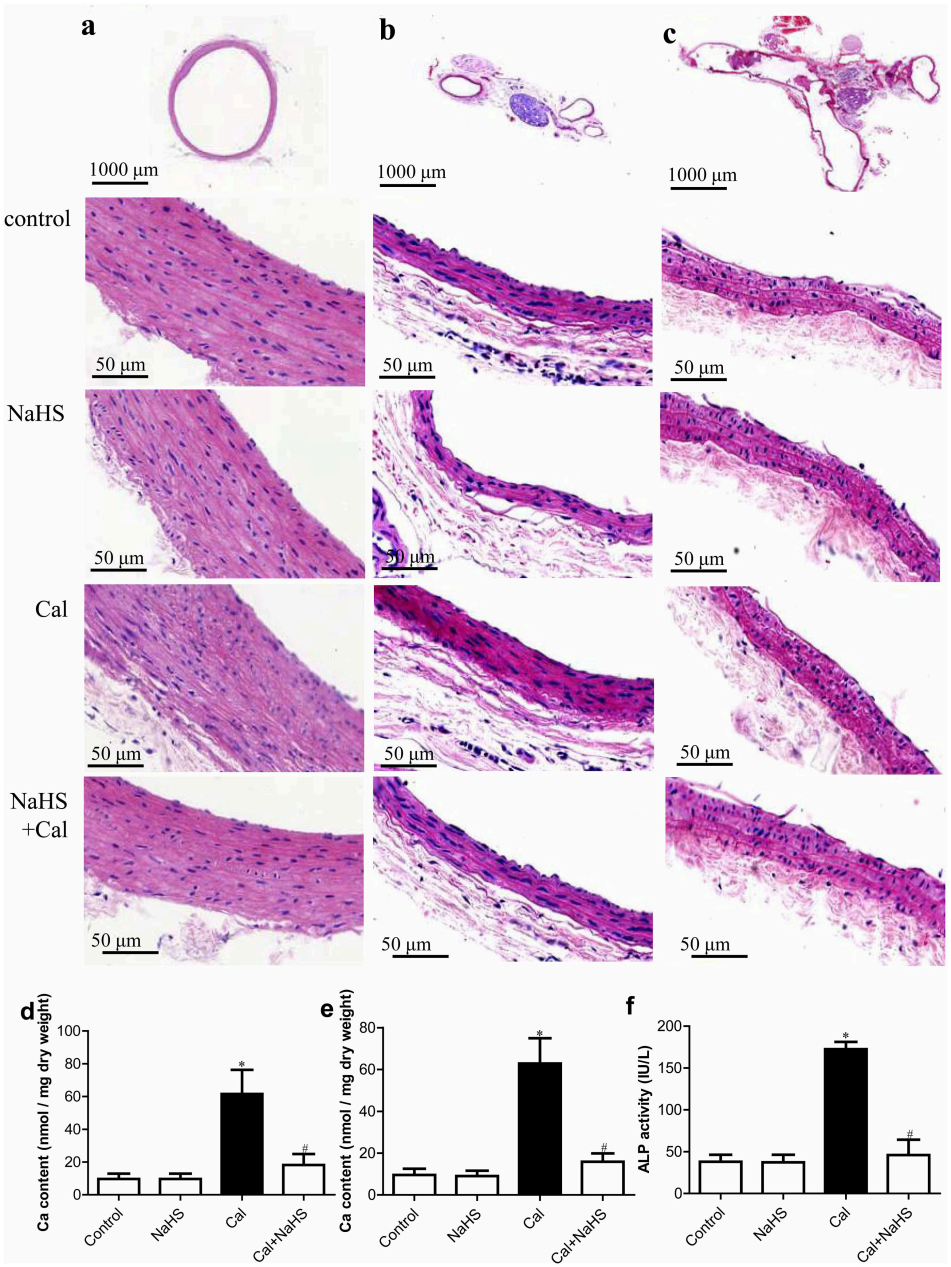
H_2_S ameliorates vascular calcification (VC) detected by morphology of vascular vessels (**a**, aorta; **b**, transverse section of carotid artery; **c**, longitudinal section of carotid artery), calcium content in aorta **(d)**, and carotid artery **(e)**, and plasma alkaline phosphatase (ALP) activity **(f)**. Data are mean ± SD. ^*^P < 0.05 vs. control; ^#^*P* < 0.05 vs. calcification (Cal) (*n* = 8 each group).

The morphology of the aorta (Figure 1a) and carotid artery (Figures 1b,c) was normal in control and NaHS treatment, but vascular elastic fibers were thickened and disordered with Cal treatment. Chronic treatment with NaHS ameliorated the VC pathology. In addition, NaHS treatment reduced the elevated Ca^2+^ content in the aorta and carotid artery and the increased plasma ALP activity in rats with VC (Figures 1d–f).

**Figure 2:**
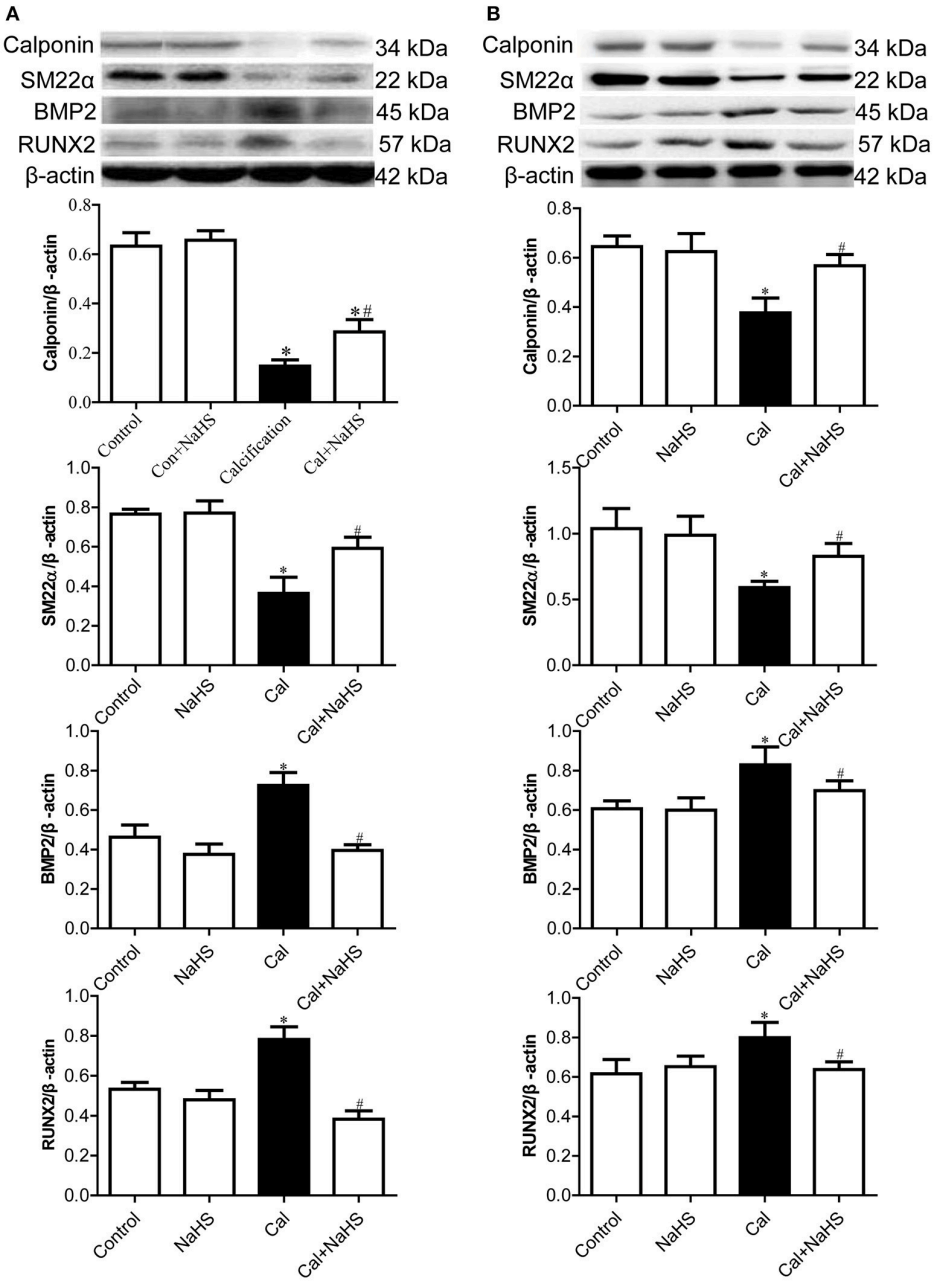
Protein expression of contractile phenotype and osteoblast-like phenotype markers of vascular smooth muscle cells in aorta **(A)** and carotid artery **(B)**. Data are mean ± SD. ^*^P < 0.05 vs. control; ^#^*P* < 0.05 vs. Cal. (*n* = 8 each group).

As compared with control and NaHS treatment, Cal treatment reduced the protein levels of the contractile phenotype markers of vascular smooth muscle cells (VSMCs), calponin and smooth muscle 22α (SM22α), in the aorta (Figure 2A) and carotid artery (Figure 2B). Chronic NaHS treatment reversed the reduced levels of calponin and SM22α. Conversely, as compared with control or NaHS treatment, Cal treatment increased the protein levels of osteoblast-like markers of VSMC, bone morphogenic protein 2 (BMP2) and runt-related transcription factor 2 (RUNX2), in the aorta (Figure 2A) and carotid artery (Figure 2B). NaHS treatment attenuated the increased levels of BMP2 and RUNX2 in calcified vessels.

### H_2_S inhibited activation of endoplasmic reticulum stress (ERS) in rats with VC

**Figure 3:**
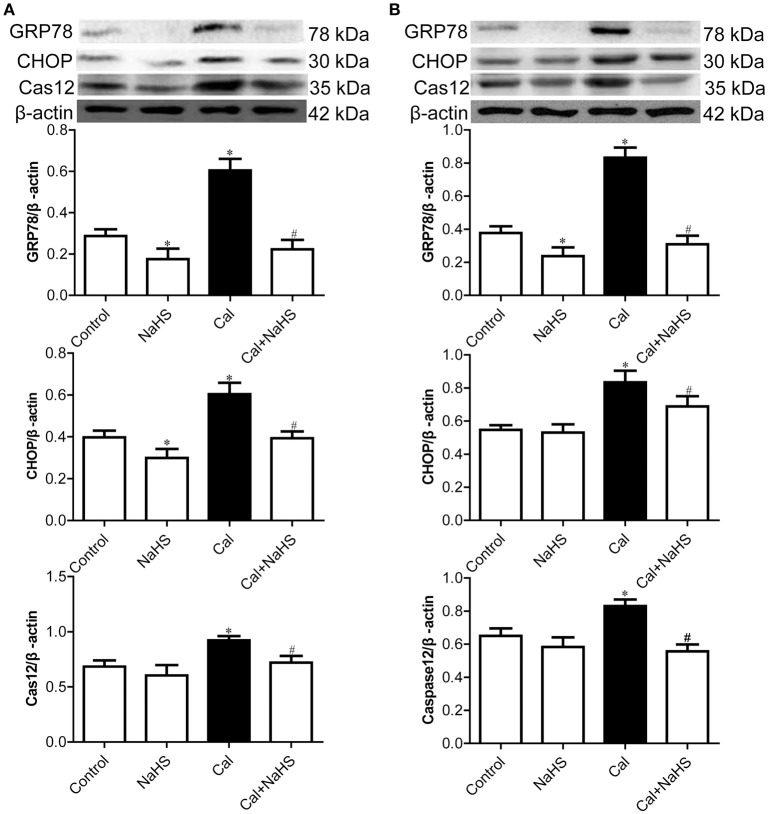
Protein expression of endoplasmic reticulum stress markers in aorta **(A)** and carotid artery **(B)**. Data are mean ± SD. ^*^P < 0.05 vs. control; ^#^*P* < 0.05 vs. Cal. (*n* = 8 each group).

To determine the effect of NaHS treatment on ERS, we detected the protein levels of general ERS markers, GRP78, CHOP and active caspase-12, both in the aorta and carotid artery by Western blot. As compared with control and NaHS treatment, Cal treatment increased the protein levels of ERS markers for VSMC, GRP78, CHOP, and active caspase-12, in the aorta (Figure 3A) and carotid artery (Figure 3B). NaHS treatment attenuated the increased levels of these proteins induced by Cal treatment both in calcified aorta and carotid artery.

### Chronic H_2_S treatment facilitated the blunted sensitivity of baroreflex in rats with VC

**Figure 4:**
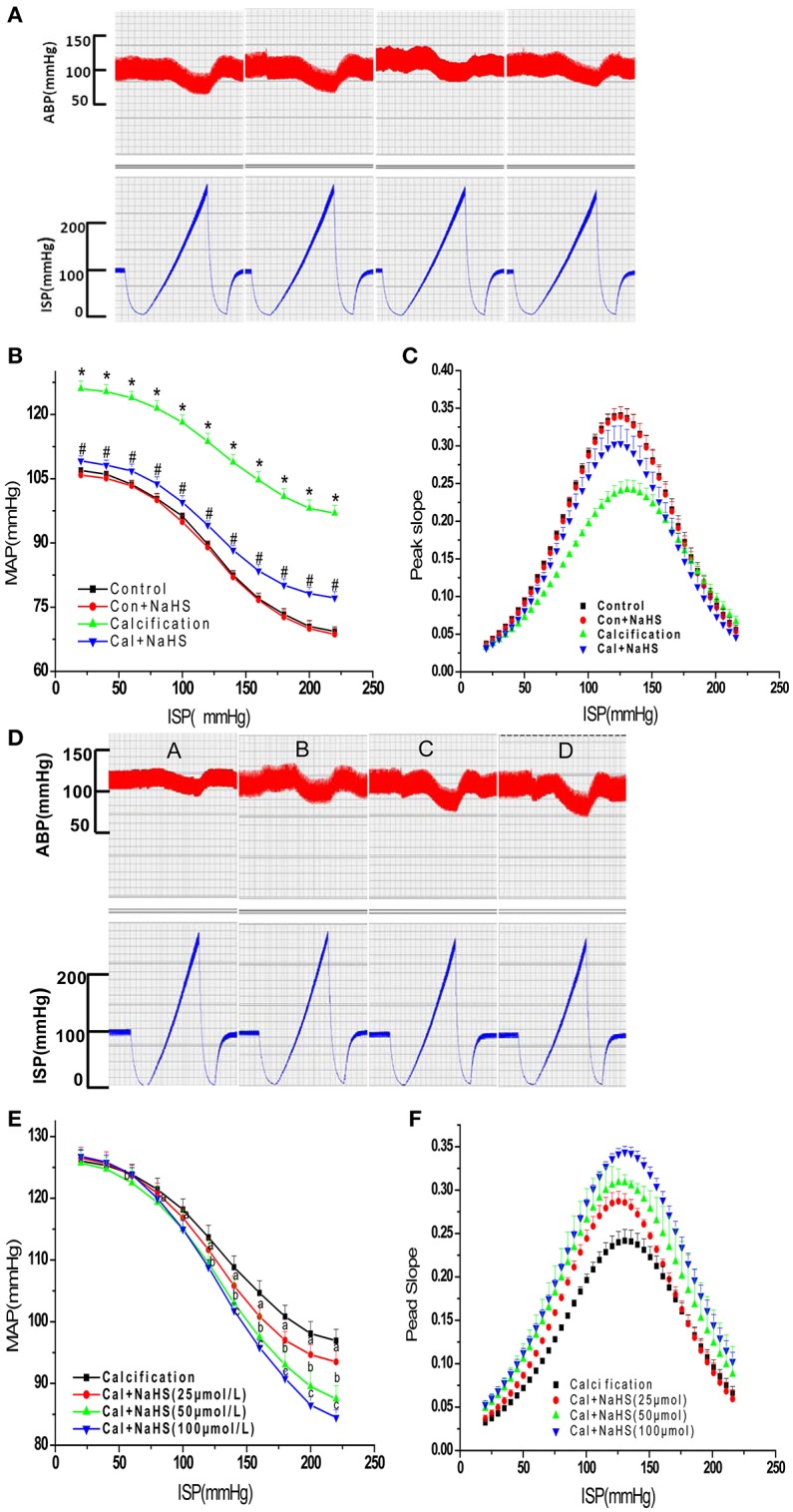
The original recording images **(A,D)**, functional curves **(B,E)** and peak slope **(C,F)** of carotid sinus baroreflex. **(A–C)**, Effect of chronic NaHS treatment on baroreflex; **(D–F)**, Effect of acute NaHS treatment on baroreflax. Data are mean ± SD. ^*^P < 0.05 vs. control; ^#^*P* < 0.05 vs. Cal. (*n* = 8 each group).

To detect the effect of NaHS on sensitivity of baroreflex, we measured the baroreflexic sensitivity by perfusion of left isolated carotid sinus on rats of control, NaHS, Cal and Cal+NaHS groups. Compared with control treatment, with Cal treatment, the functional curves of carotid sinus baroreflex were moved upward, and the peak slope was moved downward (Figures 4A–C). Consistently, TP, EP, and SP were higher with Cal than control treatment, and PS and RD were lower (Table 1). Chronic treatment with NaHS greatly ameliorated the upward movement of functional curves, the downward movement of peak slope, and the elevation of TP, EP, and SP in rats with VC.

**Table 1:** The functional parameters of carotid sinus baroreflex.

	**TP (mmHg)** **(x ± s)**	**EP (mmHg)** **(x ± s)**	**SP (mmHg)** **(x ± s)**	**PS** **(x ± s)**	**OR (mmHg)** **(x ± s)**	**RD (mmHg)** **(x ± s)**
Control	74.27 ± 0.90	96.72 ± 0.52	195.54 ± 2.82	0.34 ± 0.01	121.27 ± 3.10	37.63 ± 0.78
NaHS	74.44 ± 1.84	96.21 ± 0.76	193.03 ± 2.73	0.34 ± 0.01	118.59 ± 3.42	37.30 ± 0.99
Cal	83.32 ± 1.10^*^	114.97 ± 1.39^*^	204.25 ± 1.98^*^	0.24 ± 0.01^*^	120.94 ± 1.37	29.11 ± 0.92^*^
Cal + NaHS	78.63 ± 1.37^#^	99.56 ± 1.17^#^	201.41 ± 0.55^#^	0.30 ± 0.01^#^	122.78 ± 1.17	32.02 ± 0.77^#^

TP, threshold pressure; EP, equilibrium pressure; SP, saturation pressure; OR, operating range; PS, peak slope; RD, reflex decrease. Values are mean ± SD (n = 6).

* P < 0.05, Calcification group vs. Control group;

# *P < 0.05, Cal group*.

### Perfusion of isolated carotid sinus with H_2_S facilitated the blunted sensitivity of baroreflex in rats with VC

To determine the ameliorative effect of NaHS on sensitivity of barorelex is directed or resulted from the attenuation of VC, we valued the direct effect of NaHS on baroflex by perfusion of left isolated carotid sinus on VC rats. The carotid sinus of rats with VC was perfused with concentrations of NaHS (25, 50, 100 μmol/L). NaHS dose-dependently shifted the functional curve of the baroreflex to the left and downward, with an increase in peak slope (Figures 4D–F). Otherwise, NaHS dose-dependently decreased TP, EP, and SP and increased PS, OR, and RD in rats with Cal treatment (Table 2).

**Table 2:** The functional parameters of carotid sinus baroreflex.

	**TP (mmHg)** **(x ± s)**	**EP (mmHg)** **(x ± s)**	**SP (mmHg)** **(x ± s)**	**PS** **(x ± s)**	**OR (mmHg)** **(x ± s)**	**RD (mmHg)** **(x ± s)**
Calcification	83.32 ± 1.10	114.97 ± 1.39	204.25 ± 1.98	0.24 ± 0.01	120.94 ± 1.37	29.11 ± 0.92
NaHS (μmol/L)						
25	77.12 ± 1.34^*^	113.30 ± 1.17	201.24 ± 1.37^*^	0.29 ± 0.01^*^	124.12 ± 2.52^*^	33.18 ± 0.54^*^
50	73.94 ± 1.37^*^	112.29 ± 1.84^*^	200.57 ± 1.22^*^	0.31 ± 0.01^*^	126.13 ± 1.23^*^	38.24 ± 0.84^*^
100	69.41 ± 1.17^*^	111.46 ± 0.90^*^	197.39 ± 1.52^*^	0.34 ± 0.01^*^	122.97 ± 2.26	42.64 ± 0.73^*^

Values are mean ± SD (n = 6).

* *P < 0.05 vs. Cal group*.

## Discussion

Here, we show that NaHS treatment ameliorated thehistological disorder and Ca^2+^ content with calcification of aortas and carotid arteries in rats, inhibited the elevated plasma ALP activity, and prevented the transformation of the VSMC phenotype and activation of ERS in VC rats. Chronic NaHS treatment facilitated the blunted sensitivity of the carotid sinus baroreflex in VC rats. Acute perfusion with NaHS also dose-dependently improved the baroreflexic sensitivity in rats with VC.

Baroreflex sensitivity is considered a comprehensive marker of the universal integrity of the autonomic nervous system (Robinson and Carr, 2002). Several studies have shown reduced baroreflex sensitivity in VC (Chesterton et al., 2005), which may contribute to dysfunction of the cardiovascular system and increased mortality (McIntyre, 2007; Kaur et al., 2016). We also found that in rats with VC, the functional curve of carotid sinus baroreflex shifted upward, with reduced peak slope. These results reconfirm the blunted sensitivity of baroreflex in VC.

As an endogenous gaseous signaling molecule, H_2_S has a critical role in maintenance of cardiovascular homeostasis. Our previous study found that H_2_S facilitates baroreflexive sensitivity in normal rats (Xiao et al., 2006; Guo et al., 2011, 2016) and rescues the impaired baroreflexive sensitivity in hypertensive rats (Gu et al., 2013). The blunted baroreflexive sensitivity in diabetic rats also can be rescued by H_2_S treatment (El-Sayed et al., 2016). Here, we assessed the effect of chronic treated H_2_S on sensitivity of baroreflex. Chronic treatment with NaHS, the extensively used H_2_S donor, ameliorated the upward shift of the carotid sinus baroreflex and the reduced peak slope. Therefore, H_2_S significantly facilitates the impaired sensitivity of baroreflex in VC.

A series of investigations have shown the ameliorative effect of H_2_S on VC (Wu et al., 2006; Zavaczki et al., 2011). We previously reported that H_2_S alleviated VC in rats by preventing ERS (Yang et al., 2016). Our current data further confirm that H_2_S treatment ameliorated VC and inhibited transformation of the VSMC phenotype and activation of ERS in both the aorta and carotid artery in rats. Our results again show the protective role of H_2_S in VC.

ERS may exert crucial role in the regulation of baroreflex sensitivity. Masson et al. reported that activation of ERS is associated with autonomic dysfunction, including the impaired sensitivity of barorelex, and 4-PBA, an inhibitor of ERS, could ameliorated the impaired sensitivity (Masson et al., 2015). We also observed that NaHS inhibited activation of ERS and facilitated the baroreflex sensitivity. These results suggested that H2S might facilitated the baroreflex sensitivity via attenuation of ERS. In the future, we shall use the regulator of ERS, such as 4-PBA or tunicamycine, to confirm the speculation.

However, whether the facilitating effect of H_2_S on baroreflexic sensitivity was the direct effect or resulted from the amelioration of VC was still underlied. To detect the direct effect of H_2_S on baroreflex, we used different concentrations of NaHS perfusing the isolated carotid sinus in rats with VC. Acute treatment with NaHS dose-dependently removed the downward functional curves with elevated peak slope. These results confirm the directly facilitating effect of H_2_S on the blunted sensitivity of baroreflex.

For the mechanisms of acute treatment of NaHS facilitated sensitivity of baroreflex, the ATP-sensitive K+ channels (K_ATP_) maybe a critical mediator. Our previous articles have confirmed that H_2_S could facilitate sensitivity of baroreflex through opening K_ATP_ channels and further closing the calcium channels in vascular smooth muscle (Xiao et al., 2006). Our another experiment in spontaneously hypertensive rats (SHR) also supported that the K_ATP_ channels and the calcium channels mediated the facilitated effect of H_2_S on baroreflex in hypertension (data shown as the figures and have not published). Therefore, H_2_S might also regulate of K_ATP_ channels and subsequently close the calcium channels in rats with vascular calcification resulted in facilitating sensitivity of baroreflex. We should investigate the hypothesis in the future employed regulators of the channels or abrogation of the channels expression.

Our and other studies have reported decreased content of H_2_S and protein levels of cystathionine γ-lyase, the primary enzyme contributing to production of H_2_S in the cardiovascular system, in rats with VC (Wu et al., 2006; Yang et al., 2016). Given that H_2_S facilitates the sensitivity of baroreflex both in normal rats and rats with VC, these results suggest that the decreased levels of endogenous H_2_S in VC may be mediated by impaired baroreflexive sensitivity.

There are several limitations in our study, which should be investigated in the future. First of all, the effect of H_2_S on baroreflex should be observed in other VC models, besides the VDN model in rats, even in humans. The effect of H_2_S on the baroreflex through the central nervous system, such as rostral ventrolateral medulla, should be detected. As well, the role of impaired baroreflexive sensitivity in the pathogenesis of VC should be detected. Finally, the detailed mechanism of H_2_S-regulated baroreflexive sensitivity in VC should be investigated.

In conclusion, our results show that H_2_S could directly facilitate the impairment of baroreflex in VC and ameliorate VC. These data might provide a new target and strategy for regulating the baroreflex and therapy of VC.

## Author contributions

HL, XT, YW designed the work and analysis data. HL, XT, RY prepared the animal model. HL, QG, LX performed the perfusion of isolated carotid sinus. TY, XF performed the HE staining. XT, XD measured Ca^2+^ content and ALP activity. HL, HX performed Western blots. HL, RY, QG, LX, XF, XD, HX drafted the work, and XT, YW revised it. All authors agree to be accountable for the content of the work, and approved the final version to be published.

### Conflict of interest statement

The authors declare that the research was conducted in the absence of any commercial or financial relationships that could be construed as a potential conflict of interest.

## Abbreviations

ALP: Alkaline phosphatase
EP: equilibrium pressure
ERS: endoplasmic reticulum stress
OR: operating range
PS: peak slope
RD: reflex decrease of BP
SP: saturation pressure
TP: threshold pressure
VC: vascular calcification
VDN: vitamin D3 plus nicotine
VSMCs: vascular smooth muscle cells.
